# Late Effects of Ionizing Radiation on the Ultrastructure of Hepatocytes and Activity of Lysosomal Enzymes in Mouse Liver Irradiated In Vivo

**DOI:** 10.3390/metabo14040212

**Published:** 2024-04-09

**Authors:** Małgorzata Łysek-Gładysińska, Anna Wieczorek, Anna Walaszczyk, Karol Jelonek, Monika Pietrowska, Piotr Widłak, Roland Kulik, Dorota Gabryś

**Affiliations:** 1 Division of Medical Biology, Institute of Biology, Jan Kochanowski University, 25-406 Kielce, Poland; anna.wieczorek@ujk.edu.pl; 2 Freeman Hospital, The Newcastle upon Tyne Hospitals NHS Foundation Trust, Newcastle upon Tyne NE7 7DN, UK; anna.walaszczyk@nhs.net; 3 Center for Translational Research and Molecular Biology of Cancer, Maria Sklodowska-Curie National Research Institute of Oncology Gliwice Branch, 44-102 Gliwice, Poland; karol.jelonek@gliwice.nio.gov.pl (K.J.); monika.pietrowska@gliwice.nio.gov.pl (M.P.); 4 2nd Department of Radiology, Medical University of Gdańsk, 80-210 Gdańsk, Poland; piotr.widlak@gumed.edu.pl; 5 Department of Radiotherapy Planning, Maria Sklodowska-Curie National Research Institute of Oncology Gliwice Branch, 44-102 Gliwice, Poland; roland.kulik@gliwice.nio.gov.pl; 6 Department of Radiotherapy, Maria Sklodowska-Curie National Research Institute of Oncology Gliwice Branch, 44-102 Gliwice, Poland; dorota.gabrys@gliwice.nio.gov.pl

**Keywords:** ionizing radiation, liver, hepatocytes, lysosomes, autophagic vacuoles, lysosomal hydrolases

## Abstract

The study aimed to investigate late radiation-induced changes in the histology, ultrastructure, and activity of lysosomal enzymes in mouse liver exposed to ionizing radiation. The experiment was conducted on C57BL/6J male mice whose distal part of the liver was exposed occasionally to single doses of radiation (6 MV photons) during targeted heart irradiation; estimated doses delivered to analyzed tissue were 0.025 Gy, 0.25 Gy, 1 Gy, and 2 Gy. Tissues were collected 40 weeks after irradiation. We have observed that late effects of radiation have an adaptive nature and their intensity was dose-dependent. Morphological changes in hepatocytes included an increased number of primary lysosomes and autophagic vacuoles, which were visible in tissues irradiated with 0.25 Gy and higher doses. On the other hand, a significant increase in the activity of lysosomal hydrolases was observed only in tissues exposed to 2 Gy. The etiology of these changes may be multifactorial and result, among others, from unintentional irradiation of the distal part of the liver and/or functional interaction of the liver with an irradiated heart. In conclusion, we confirmed the presence of late dose-dependent ultrastructural and biochemical changes in mouse hepatocytes after liver irradiation in vivo.

## 1. Introduction

The liver is responsible for the broad and multidirectional involvement in maintaining the homeostasis of organisms [1,2]. It is a multifunctional organ with a hierarchical structure consisting of multiple hepatic lobules that act as functional units of tissue. Of all the cells that make up liver tissue, hepatocytes constitute the majority: approximately 80% of the cells that are functionally responsible for carbohydrate metabolism, protein synthesis, detoxification of xenobiotics, and also participate in the process of bile secretion [3,4].

Metabolic processes occurring in living cells and tissues depend on the rate of synthesis and degradation of basic energy compounds. These processes are largely regulated by hydrolytic lysosomal enzymes such as phosphatases, proteases or glycosidases, and others [5,6]. In order to maintain cellular homeostasis, lysosomal enzymes degrade material of extracellular and intracellular origin in ATP-independent processes in mostly normal cells of various types of tissues, as well as in cells exposed to various damaging factors such as hypoxia or lack of nutrients [7,8]. Intracellular homeostasis of hepatocytes can be compromised in many tissue injuries, including cardiac dysfunction caused by ionizing radiation. According to numerous studies, ionizing radiation can lead to labilization of lysosomal membranes and increased activation of enzymes that degrade damaged cell organelles [9,10,11]. As we know, the goal of radiation therapy is to kill cancer cells, but with as little damage as possible to healthy tissue [12]. Unfortunately, tissue irradiation is inextricably linked to the occurrence of early and late radiation side effects [13,14,15]. Thus, an important problem of radiotherapy used to treat cancer is also the response of healthy cells. Early effects occur during or shortly after completion of radiotherapy; late effects that manifest 6 months to several years after radiotherapy include fibrosis, atrophy, vascular damage, and hormone deficiencies [13]. They are usually irreversible and potentially progressive [16]. The late radiation response involves the interaction of multiple intercellular communication pathways that involve the release of growth factors and pro-inflammatory cytokines that stimulate the adaptive response of cells involved in wound healing [17,18].

Modern methods used in radiation therapy make it possible to irradiate the tumor volume with ionizing radiation, saving the volume of healthy tissues to a greater extent than using older techniques [19,20,21]. Despite technological advances, we are still not able to completely protect healthy tissue, which is still a limitation during radiation therapy. Thoracic radiation has been associated with a significantly increased risk of unintentional irradiation of the heart and induction of radiation-induced heart disease (RIHD) and dysfunction of the cardiovascular system [22]. Exposure of the heart during thoracic irradiation occurs especially during the treatment of breast and lung cancers, and mediastinal lymphomas. Even low doses unintentionally administered to the heart (0.5 Gy) can significantly enhance the risk of late cardiovascular complications [23]. It is known that the liver and heart are anatomically and physiologically connected primarily via the vascular system. Therefore, it is important to study complex cardio–hepatic interactions, especially when one of the organs is exposed to pathological factors. The presented work focuses on late post-radiation changes in the liver, resulting from the functional interaction between the heart and liver, as well as a result of unintentional irradiation of the distal part of the liver during targeted cardiac irradiation. In the case of radiotherapy of the right breast, due to anatomical proximity, the liver is one of the normal tissues exposed to radiation and the risk of damage, the etiology of which may be multifactorial [24]. Knowing the ionizing radiation-induced pathological changes in the liver is important to ensure safe and effective radiation therapy.

In our project, during cardiac irradiation in mice, a substantial part of the liver is also irradiated, which can induce pathological changes in the structure of hepatocytes and the activity of its enzymes. Therefore, this study investigated the long-term effect of indirect effects of low and high doses of ionizing radiation applied to the heart (0.2 Gy, 2 Gy, 8 Gy, and 16 Gy), which corresponds to mean doses to the distal part of the liver of 0.025 Gy, 0.25 Gy, 1 Gy, and 2 Gy, respectively, on histology, ultrastructure, and activity of lysosomal enzymes in mouse liver.

## 2. Materials and Methods

### 2.1. Animals and Irradiation Procedure

Male C57BL/6J mice were purchased from Charles River Laboratories (Research Models and Services Germany GmbH, Sulzfeld, Germany). The age of the animals at the time of irradiation was 8 ± 1 weeks. The animals were kept at room temperature (21 °C) in naturally controlled light and dark 12:12 and were given laboratory food ad libitum as previously reported [25]. In each group, we included 10 animals. Before conducting our experiments, representative mice were scanned in the irradiation position with CT, and using the Eclipse system, the heart, lung, and liver were visualized and contoured. Treatment plans were performed to calculate the exact number of MU needed to deliver the required dose to the heart when 50% of the dose was given from the 0° field and 25% from the side fields. The constant source-to-surface distance (SSD) was set to 100 cm and information obtained from treatment plans allowed to create an optimal dimension of three treatment fields of 1.5 × 1.5 cm. Irradiation was performed under Avertin anesthesia in our center using Varian CLINAC 2300 linear accelerator, with 6MV photons, the dose rate was set to 300 MU/min as previously reported [26].

Single local heart doses of X-ray radiation (0.2 Gy, 2 Gy, 8 Gy, or 16 Gy) were applied. The exact position of the heart was assessed by radiography before irradiation. Livers were unintentionally exposed to irradiation during the irradiation experiment of the whole heart volume. In the case of whole heart irradiation with a dose of 8 Gy, the mean dose delivered to the whole liver was about 4.9 Gy (50% of the liver volume was irradiated with a dose of 4 Gy and higher), while 10–15% of the liver volume was irradiated with doses below 1 Gy. The distal parts of liver used here for experiments were exposed to the approximated mean dose of 1 Gy. Other single doses of 0.2 Gy, 2 Gy, and 16 Gy to the heart correspond to approximated mean doses to the liver of 0.123 Gy, 1.23 Gy, 9.8 Gy, and mean doses to the resected distal parts of 0.025 Gy, 0.25 Gy, and 2 Gy, respectively.

Mice in the control group were “sham-irradiated” in parallel to experimental animals. At 40 weeks after irradiation, the animals were sacrificed by cervical dislocation. Livers were removed and processed immediately after the decapitation of animals.

### 2.2. Analysis of Morphology

Immediately after resection of the distal part of the liver lobe, it was cut into suitable pieces (2 mm^3^) and fixed by immersion in buffered 3% glutaraldehyde in 0.1 M cacodylate buffer (pH 7.2) for at least 2 h at 4 °C. The tissue samples were then post-fixed in 2% osmium tetroxide in 0.1 M cacodylate buffer (pH 7.2) for 1 h at 20 °C. The fixed tissues were performed in a graded series of ethanol and then transferred into mixture of ethanol and propylene oxide and then in pure propylene oxide. The samples were then immersed in a series of mixtures of Epon 812 resin mixtures (Agar Scientific Ltd., Stansted, UK) and propylene oxide. Finally, the liver samples were embedded in pure liquid Epon 812 [27]. Semi-thin (500 nm) and ultra-thin (40–60 nm) sections were prepared using a Reichert-Jung ultramicrotome (Reichert, Vienna, Austria). Semi-thin sections were stained with toluidine blue and examined using a light microscope ZEISS Axio Scope.A1 with a black and white digital camera (Oberkochen, Germany). Representative fields from each slide were photographed and acquired with the photo-image capture software. Ultrathin sections were stained with uranyl acetate and lead citrate. Ultrastructure evaluation was performed using a transmission electron microscope Tesla BS500 with Frame Transfer-1K-CCD-Camera (TRS, Moorenweis, Germany).

Morphometric analysis of 50 randomly selected electron micrographs from each group of animals was performed to evaluate the average number of lysosomes and autophagic vacuoles.

Data were analyzed by the nonparametric Mann–Whitney U test using STATISTICA software (Ver. 10. StatSoft Company, 2011, Hamburg, Germany). *p* < 0.05 was considered a significance threshold.

### 2.3. Analyses of Lysosomal Enzymes Activity

Immediately after resection, liver tissue was homogenized in a medium consisting of 0.25 M sucrose in a Potter–Elvehjem glass homogenizer (in the proportion: 1 g of tissue per 7 mL sucrose) with a Teflon piston operated at 200 rpm according to the modified method of Marzella and Glaumann [28]. The homogenates were fractionated by differential centrifugation. The first centrifugation was performed at 1000× *g* for 10 min to remove cell debris, nuclei, and heavy mitochondria. The resulting supernatant was centrifuged at 20,000× *g* for 20 min; the resulting pellet, that is, the “lysosomal fraction”, contained a mixture of lysosomes (the dominant component), light mitochondria, peroxisomes, and the endoplasmic reticulum. The pellet was suspended in 5 mL of 0.1% TRITON X-100 to release latent lysosomal enzymes. The pellet was then frozen and stored at −20 °C until analysis. For this, the samples were thawed, transferred to 1.5 mL Eppendorf tubes, and centrifuged at 12,000× *g* for 2 min to remove debris and insoluble material.

Acid phosphatase activity [AcP, EC 3.1.3.2] was determined according to the method of Hollander [29], while the activity of β-glucuronidase [BGRD, EC 3.2.1.31], N-acetyl-β-D-hexosaminidase [HEX, EC 3.2.1.30], and β-galactosidase [BGAL, EC 3.2.1.23] was determined according to the method of Barrett [30]. The activity of arginine aminopeptidase [ARG, EC 3.4.11.6] was determined by the method of McDonald and Barrett [31]. The total activity of cathepsins D [EC 3.4.23.5] and L [EC 3.4.22.15] was assessed according to Langner et al. [32]. The total protein level was determined by a modification of the Lowry method [33]. Enzyme activity was expressed in µM of products per mg of total protein per hour. The absorbance was measured with the use of a Spekol 1500 UV/VIS spectrophotometer (Analityk Jena AG, Jena, Germany). The statistical significance of the differences was evaluated using the student’s *t*-test for unpaired data using the Statistica software (Ver.10. StatSoft Company, 2011).

The number of lysosomes and autophagic vacuoles per cell were calculated for each animal group. The data were analyzed by the nonparametric Mann–Whitney U test using the Statistica software (Ver. 10. StatSoft Company, 2011) and *p* < 0.05 was considered a significance threshold. We conducted one-way ANOVA tests with Tukey’s multiple comparisons test (95% CI of diff.), using GraphPad Prism, version 6 (GraphPad Software, San Diego, CA, USA).

## 3. Results

**Figure 1:**
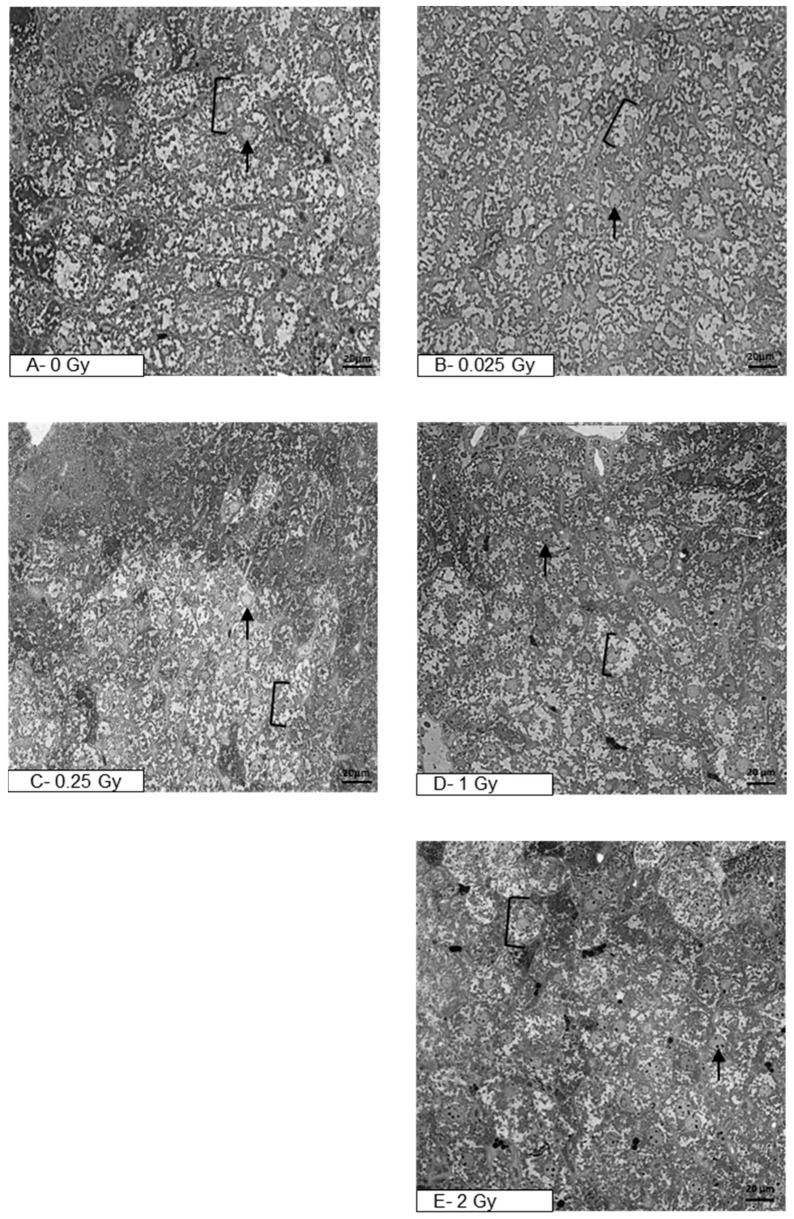
Photomicrographs of semi-thin sections of mouse liver after staining with toluidine blue (400×, light microscope). The figure shows representative pictures of the liver tissue of sham-irradiated mouse (**A**) and liver tissue after organ irradiation with single dose of 0.025 Gy (**B**), 0.25 Gy (**C**), 1 Gy (**D**), or 2 Gy (**E**). Clamps indicate hepatocytes, black arrows—nucleus (N); scale bar = 20 μm.

The delayed effect of ionizing radiation on the mouse liver tissue after prior direct heart exposure was analyzed in semi-thin sections under light microscopy (Figure 1A–E). Figure 1A presents a section of liver tissue of a control mouse (sham-irradiated, 0 Gy), characterized by normal hepatocytes which maintain a polygonal morphology with distinct cell boundaries. Hepatocytes have a round vesicular nucleus with one or a few nucleoli. Figure 1B–E present liver tissue after a single liver irradiation with doses 0.025 Gy, 0.25 Gy, 1 Gy, or 2 Gy, respectively. Semi-thin sections reveal hepatocytes with distinctly stained nuclei of various shapes. The most common are cells having one, or sometimes two, large round to slightly oval nuclei. Cells with smaller, dark-stained oval nuclei are also detected. In summary, the liver structure of irradiated animals corresponds to the morphology of the control liver tissue.

**Figure 2:**
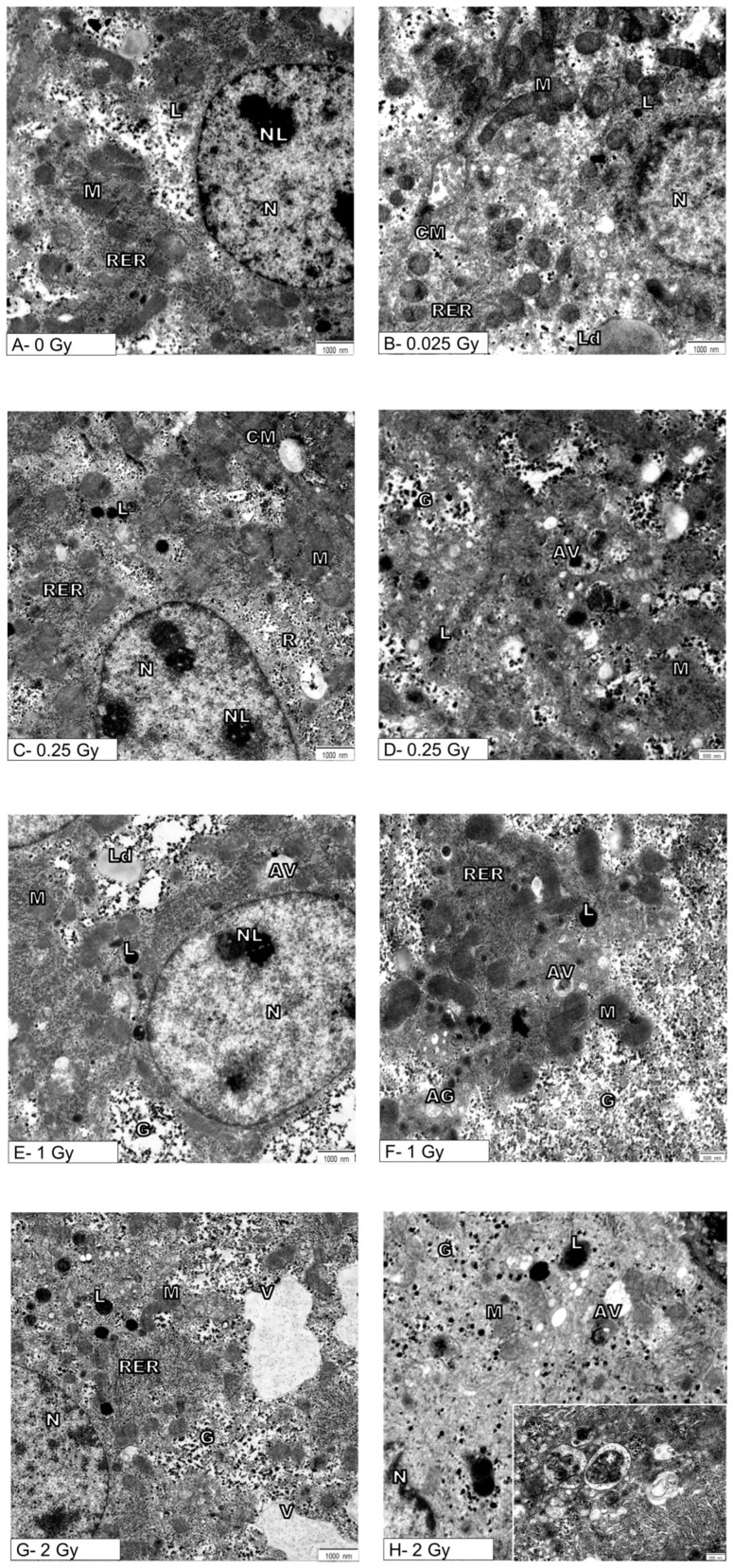
Effects of exposure to ionizing radiation on the ultrastructure of mouse hepatocytes. Electronograms show representative pictures of hepatocytes of sham-irradiated liver (**A**) and hepatocytes after organ irradiation with single dose of 0.025 Gy (**B**), 0.25 Gy (**C**,**D**), 1 Gy (**E**,**F**), or 2 Gy (**G**,**H**). N—nucleus, NL—nucleolus, M—mitochondria, RER—rough endoplasmic reticulum, AG—Golgi apparatus, L—lysosomes, CM—cell membrane, Ld—lipid droplets, AV—autophagic vacuoles, V—vacuoles, G—glycogen. Inset: autophagic vacuoles at high magnification. Scale bar = 1000 nm (**A**,**C**,**E**,**G**), scale bar = 500 nm (**D**,**F**,**H**).

More details on cellular changes induced by the indirect effect of ionizing radiation were provided by ultrastructural analysis of hepatocytes (Figure 2A–H).

Figure 2A shows the ultrastructure of the hepatocytes from the control mouse (0 Gy). Namely, the spherical or oval cell nucleus is located centrally in the hepatocyte. It is characterized by a typical pattern of nuclear architecture in which euchromatin is located mainly in the center of the nucleus, heterochromatin is located in close proximity to the nuclear envelope and also forms clusters throughout the nucleoplasm. The double membrane of the nuclear envelope, including the perinuclear space and nuclear pores, is clearly visible without signs of damage. The cytoplasm is rich in normal mitochondria whose inner membrane occasionally forms the laminar cristae and surrounds the normal-density mitochondrial matrix. The canals of the rough endoplasmic reticulum are evenly arranged near the mitochondria. Evenly distributed glycogen rosettes and ribosomes are also visible in the cytoplasm. Also, single small primary lysosomes are visible near the nucleus and bile canaliculus.

After ionizing radiation at a dose of 0.025 Gy, cells containing normal cell nucleus (N) with a nucleolus (NL) were found. Mitochondria (M) retained their normal shape and showed no visible swelling, and normal canals of the rough endoplasmic reticulum were present. There were also single primary lysosomes and lipid droplets in the cytoplasm that are typical of a normal cell (Figure 2B).

**Figure 3:**
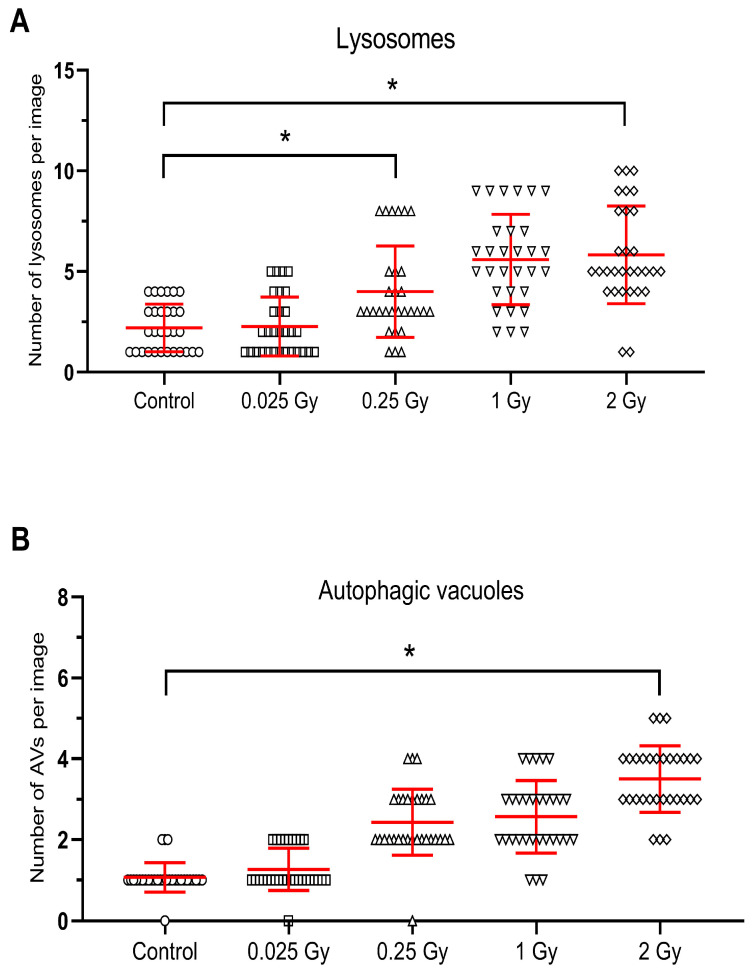
Radiation-induced increase in the number of lysosomes (**A**) and autophagic vacuoles (**B**) in hepatocytes after untargeted liver irradiation. Significant differences between compared groups are marked with asterisks: * *p* < 0.05. Raw results were plotted on the graphs.

Ionizing radiation at a dose of 0.25 Gy did not cause changes in the cell nucleus, and the mitochondria (M) retained the correct mitochondrial cristae and matrix (Figure 2C,D). There were no significant changes in the endoplasmic reticulum (RER) and the Golgi apparatus, which showed the characteristics of a normal structure. However, a statistically confirmed increase in the number of primary lysosomes (L) and autophagic vacuoles (AV) is demonstrated in Figure 2C,D and Figure 3A,B.

After untargeted liver irradiation with a dose of 1 Gy (8 Gy to heart), significant changes were found in the ultrastructure of the hepatocyte compared to the control hepatocyte Figure 2E,F. Damaged mitochondria (M) and swollen cisterns of the Golgi apparatus (AG) (single scattered bubbles) were visible. An increase in the number and size of primary lysosomes (L) and autophagic vacuoles (AV) was found (Figure 2E,F and Figure 3A,B). The structure of the nucleus (N) and nucleolus (NL) was normal with a slightly swollen nuclear envelope.

The greatest changes at the ultrastructural level of mouse hepatocytes compared to the control cells were observed after liver irradiation with a dose of 2 Gy (Figure 2G,H). In hepatocyte-damaged mitochondria, numerous autophagic vacuoles containing degradative material and numerous primary lysosomes were found (Figure 2G,H). Attention was drawn to the large bright/empty spaces bounded by the membrane—vacuoles (V) (Figure 2G). The structure of the cell nucleus (N) and nucleolus (NL) appeared normal with a slightly swollen nuclear envelope.

Statistical analysis confirmed an increase in the number of primary lysosomes after 0.25 Gy and 2 Gy and autophagosomes after 2 Gy radiation doses to the liver (Figure 3A,B).

Although the ultrastructure of hepatocytes exposed to 1 Gy and 2 Gy showed the presence of damaged mitochondria, the Mann–Whitney U test did not confirm the significance of the relationship (Figure 2).

**Figure 4:**
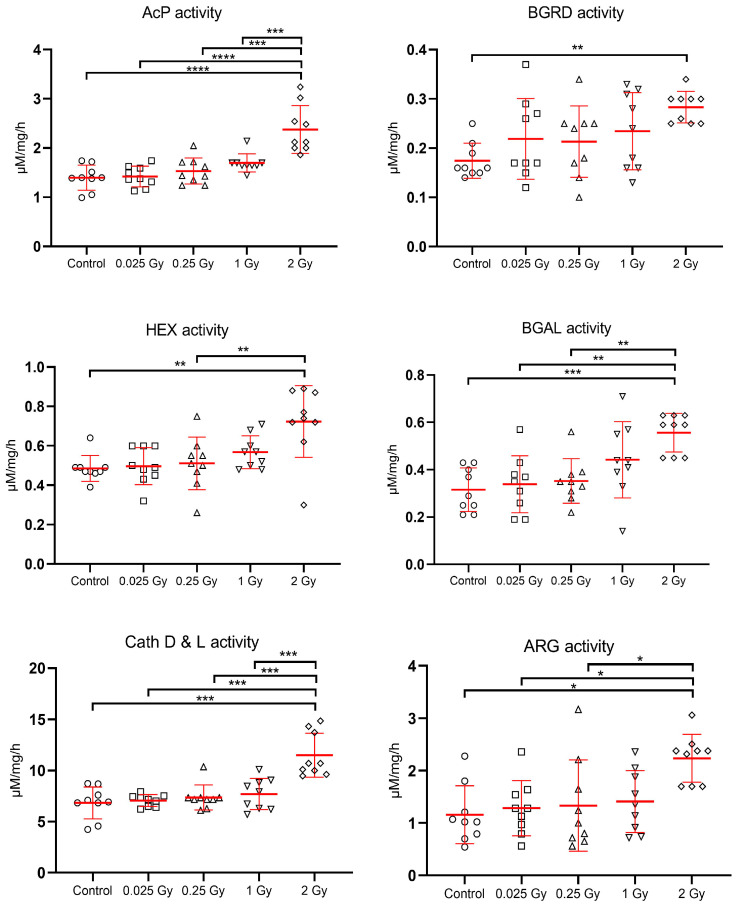
Effects of indirect exposure to ionizing radiation on the activity of liver lysosomal enzymes [expressed in µM/mg protein/hour]; mean values ± SD; asterisks represent statistical significance of changes (* *p* < 0.05, ** *p* < 0.01, *** *p* < 0.001,**** *p* < 0.0001).

The late indirect effect of ionizing radiation on the activity of lysosomal enzymes in the lysosomal fraction of liver cells is shown in Figure 4. In general, the indirect exposure of liver as a consequence of cardiac irradiation resulted in an increase in the activity of estimated liver enzymes relative to control values (control mice sham-irradiated).

The highest increase in the activity of liver lysosomal enzymes was observed after a dose of 2 Gy to the liver. All enzymes tested, especially cathepsins D and L increased their activity significantly (*p* < 0.001) in response to this dose. Lower doses of radiation also induced an increase in the activity of the hydrolases tested, but it was not significant in relation to the control. There was a significant increase in enzyme activity after irradiation with a dose of 2 Gy compared to treatment with very low doses: 0.025 Gy, 0.25 Gy, 1 Gy for AcP and Cath D&L; vs. 0.025 Gy, 0.25 Gy for BGAL and ARG, and for HEX vs. 0.25 Gy (Figure 4).

## 4. Discussion

The liver is responsible for the metabolism of carbohydrates, proteins, fats, the biosynthesis of bile acids, and the biodegradation of bioactive substances such as hormones. Its function depends on the type and dynamics of the disease process that takes place in the cardiovascular system [34,35,36]. Therefore, cardiac irradiation and the occurrence of complications in the cardiovascular system may influence liver complications. One of the adverse effects of the use of ionizing radiation within chest is an increased risk of inducing heart disease or exacerbating existing conditions [37,38]. The increased likelihood of cardiovascular disease is due to the increased life expectancy of oncology-treated patients as a result of the increasing efficacy of current therapies [39]. Cardiac abnormalities that occur many years after radiation therapy, such as arrhythmias, ischemia, inflammatory changes in the myocardium or pericardium, and sometimes acute heart failure, also adversely affect liver function [37,40,41,42].

Ionizing radiation generates reactive oxygen species, which are the main cause of damage to lysosomes, including labilization of lysosomal membranes and the release of lysosomal enzymes, which can consequently lead to severe cell damage and even cell death [11,43,44,45,46]. This can lead to structural and functional changes at the cellular level including damage to the cell membrane, cell nucleus, mitochondria, Golgi apparatus, or peroxisomes [9,44,47].

In our study, there was an increase in the activity of estimated liver enzymes relative to control values (control sham-irradiated mice). The highest increase in activity was observed after a mean dose of 2 Gy to the analyzed distal liver (16 Gy to the heart). All enzymes tested, especially cathepsins D and L, significantly increased their activity in response to this dose. This did not correspond to histological evaluation, since no significant changes in mouse liver were found 40 weeks after cardiac irradiation with single low and high mean doses of 0.25 Gy, 2 Gy, 8 Gy, or 16 Gy (vs. analyzed distal liver 0.025 Gy, 0.25 Gy, 1 Gy, or 2 Gy, respectively). On the contrary, ultrastructural analysis of hepatocytes performed using transmission electron microscopy (TEM) showed late and indirect effects of ionizing radiation on their ultrastructure. The intensity of damage increased with dose and involved an increase in the number of primary lysosomes and autophagic vacuoles. However, the greatest changes in the ultrastructure of hepatocytes were observed after a dose of 2 Gy, also the activity of lysosomal enzymes increased in proportion to the applied radiation dose.

Radiobiologists are constantly trying to locate the primary damage caused by ionizing radiation in living organisms. Although studies focus mainly on DNA damage and repair mechanisms, numerous studies show that cytoplasmic organelles are also involved in the mechanism of cell damage after irradiation [17,44]. Also, lysosomes appear to be an important cytoplasmic target for ionizing radiation [9,43]. One of the primary mechanisms of cell damage and death after radiation exposure is the uncontrolled release of acid hydrolases from the lumen of the lysosome into the cytosol, their activation from a latent state, and their destructive effects on essential cytoplasmic structures [45,48]. The ultrastructure of hepatocytes exposed to 1 Gy and 2 Gy showed the presence of damaged mitochondria, but there was no significant increase in the number of damaged mitochondria. The results presented here are supported by results from our previous work where similar ultrastructural changes in hepatocytes were shown 60 weeks after cardiac radiotherapy with a dose of 8 Gy [49].

Some biological systems maintain the normal physiology in response to low or mild stress caused by chemical or physical stimuli, such as radiation. These include antioxidant systems, which are particularly active in detoxifying organs such as the kidneys, lungs, and liver, as well as the vascular system. Numerous works have shown that low doses of ionizing radiation induce adaptive changes in the cell [50,51,52]. The results of our study are also consistent with this hypothesis. After 40 weeks of distal part liver irradiation with a mean dose of 0.025 Gy, no significant histological, ultrastructural, and biochemical changes were demonstrated in the mice liver. Low-dose radiation (<0.5 Gy) exposures can be used as an experimental tool to induce bioresponses such as the bystander effect, adaptive response, and genomic instability. The distinct biological effects of low-dose radiation can be seen to be different in normal and cancer cells [53,54]. The adaptive response mechanisms include gene transcription, the activation of diverse signaling and stress response pathways that trigger cell defenses such as enhanced DNA repair systems, the induction of protein synthesis, enhanced detoxification free radical and antioxidant production, cell survival/death pathway (apoptosis), cytoprotective processes including autophagy, protein unfolding, and enhanced immune response [54,55].

Research shows that the effect of post-radiation changes in cells depends on the passage of time after radiation therapy. The study by Telbisz et al. [43] showed a significant increase in the number of autophagic vacuoles and the number of apoptotic cells in vivo in pancreatic cells after a dose of 8 Gy, a few hours after irradiation, but no such changes were detected after a longer period. In the work of Wieczorek et al. [56], an indirect effect of ionizing radiation on the liver of mice was already demonstrated several hours (12–120 h) after irradiation of the distal liver with a mean dose of 1 Gy. The ultrastructural image of mouse hepatocytes showed an increase in the number of autophagic vacuoles and an increased activity of lysosomal hydrolases, but autophagy was not confirmed. Our late effect of ionizing radiation on the ultrastructural profile of mouse hepatocytes and the reactivity of the lysosomal system, presented in this paper, clearly showed the largest significant changes after the highest applied dose of 2 Gy to the resected distal part of the liver. Moreover, as numerous studies show, radiation-caused damage to hepatocytes and the changes in reactivity of the lysosomal system strictly depend on the genetic conditions of the organism, radiation dose, and time after radiotherapy [34]. The work of Lysek-Gladysinska et al. [49] showed greater potential damage to hepatocytes in Apo E deficiency mice genetically burdened with cardiovascular diseases than in wild-type mice.

The data presented in this study confirm the appearance of ultrastructural and biochemical changes in mouse hepatocytes, probably caused by a cellular adaptive response to local liver irradiation, and confirm the functional relationship between the irradiated heart and the liver. Considering the strong functional connection between the liver and the cardiovascular system, the impact of radiation damage to the heart directly exposed to high doses of radiation (ranging from 0.2 Gy to 16 Gy) on the changes observed in the liver should be taken into account. Over longer periods, the occurrence of changes may reflect the functioning of the entire liver and the fact that a large part of it was exposed to higher dose irradiation. The analysis of the ultrastructure of the hepatocyte carried out in a transmission electron microscope provides detailed information about changes in individual cell organelles and combined with the analysis of lysosomal enzyme activity gives a broader picture of pathological cellular changes.

## 5. Conclusions

Long-term effects of radiation observed in the ultrastructure of hepatocytes 40 weeks after exposure of the liver included a dose-dependent increase in the number of primary lysosomes and autophagic vacuoles, which were noted at doses as low as 0.25 Gy and higher doses. The most important biochemical effect was the increase in all lysosomal hydrolases activity in tissues exposed to 2 Gy.

## Data Availability

Data presented in this study are contained in this article, or available upon request to the corresponding authors.
